# Validation and Application of Screen-Printed Microchip for Potentiometric Determination of Metformin Hydrochloride in Tablet Dosage Form

**DOI:** 10.1155/2024/8664723

**Published:** 2024-02-27

**Authors:** Mohammed Alqarni, Abdullah A. Alshehri, Hassan Arida

**Affiliations:** ^1^Department of Pharmaceutical Chemistry, College of Pharmacy, Taif University, P.O. Box 11099, Taif 21944, Saudi Arabia; ^2^Department of Clinical Pharmacy, College of Pharmacy, Taif University, P.O. Box 11099, Taif 21944, Saudi Arabia

## Abstract

Metformin is an oral biguanides hypoglycaemic agent, which used to lower the blood glucose levels in people with type 2 diabetes mellitus. Many analytical techniques have been used to quantify the drug in different pharmaceutical dosage forms; however, most of these methods have limited throughput in the quality control application. A disposable potentiometric microsensor responsive to metformin has recently been reported. For the first time, herein, this method of analysis has been validated according to IUPAC recommendations and successfully applied in the determination of metformin drug in some dosage form. Different drug formulations of metformin hydrochloride have been collected from the local pharmaceutical stores in Saudi Arabia and analysed using the validated microchip-based method of analysis. Subsequently, the results of this study showed that the validated method was linear, specific, precise, and accurate. The linear range was 1 × 10^−1^–1 × 10^−5^ mol L^−1^ and the correlation coefficient was 0.999. The limit of detection was 2.89 × 10^−6^ mol L^−1^, and the limit of quantification was 8.77 × 10^−6^ mol L^−1^. This method demonstrated high precision, with an RSD% of less than 2.22%. The accuracy of this method was obtained by comparing the recovery percentage with percentage values less than 5%. The results obtained showed that there was no significant difference between the references, label, and recovery of less than 5%.

## 1. Introduction

Metformin (MTE) is the most frequently prescribed oral medication as a treatment for people with type 2 diabetes. It lowers blood glucose levels and increases insulin sensitivity in the body, preventing potential diabetic complications such as eye damage, kidney damage, nerve damage, and sexual dysfunction [1–5]. MET is also a preferred antidiabetic drug due to its high efficacy, good safety profile, and low cost [6]. Furthermore, considerable effectiveness of MET on obesity [7], cardiovascular diseases [8], liver diseases [9], cancers [10, 11], and renal diseases [12] were reported. MET hydrochloride (also known as N, N dimethyl imido dicarbonimidic diamide hydrochloride) has the empirical formula C4H11N5.HCl and a molecular weight of 165.63 g/mol. Subsequently, because of its ubiquitous usage, continuous monitoring of MET levels in pharmaceutical formulations and in human plasma has long been a crucial concern. Since the quality of pharmaceutical formulations generally determines the efficacy and safety of MET treatment.

MET quality control (QC) generally requires an assay with a high throughput capability. For the purpose of determining MET, several instrumental techniques have been developed [13]. These techniques include high-performance liquid chromatography [14, 15], UV-visible spectrophotometry [16–18], LC-MS/MS [19, 20], electrochemical methods of analysis [21–25], spectrofluorimetric methods [26], and varied HPTLC techniques [27–29]. Spectrophotometric assays are considered practical procedures due to their high sensitivity, simplicity, low cost, and wide accessibility in laboratories. However, a majority of these assays have substantial limitations, such as low selectivity because their measurements are made in the UV region [30–32], decreased assay procedure simplicity, and laborious liquid–liquid extraction stages [33–35]. Furthermore, due to differences in the chemical structures of MET, these assays were developed individually. Thin-film microelectrode development, on the other hand, has recently received more interest than previous techniques due to its inherent simplicity, high sensitivity, quick analysis, low cost, large-scale production, and automated and integrated feasibility [36–42].

Consequently, scientists and researchers have been developing analytical techniques with high-throughput capacities to increase the QC analysis and improve its productivity. High-throughput assays enable researchers to efficiently process massive quantities of samples; hence, uniformity of pharmaceutical formulations, rapid identification of active substances, and other pharmaceutical industry operations which could be achieved. Recently, Alfadhel et al. [23] fabricated a novel disposable microchip that demonstrated significant reliability, good credibility, low cost, and rapid determination of MET. Therefore, this research aimed to validate and investigate the realized potentiometric microsensor for the QC application of MET for the first time.

## 2. Materials and Methods

### 2.1. Apparatus and Tools

**Figure 1:**
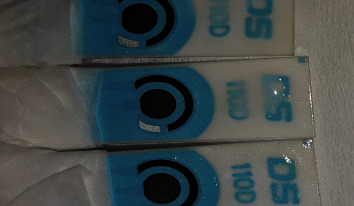
Photographic picture of fabricated screen-printed microchip assemblies [23].

A Jenway (model 3510) pH/mV meter and Jenway combination pH electrode for all pH experiments were used for electrochemical characterization measurements. The metformin-based microchip (Figure 1) has been fabricated, characterized, and used in the metformin analysis as described in our previous work [23]. For MET detection, the microchip was used as the working electrode which based on a tetraphenyl borate/MET ion pair modified with carbon nanotubes in conjunction with the reference electrode (metrohm double junction electrode), as mentioned in the previous teamwork [23]. Double-distilled water was obtained from an Aquatron water distiller (A4000D, Bibby Scientific, UK, 1.0 MΩ cm^−1^), and it was used to prepare the samples and rinse the glassware.

### 2.2. Standards Pharmaceutical Formulation and Reagents

The MET hydrochloride raw material (purity: 99.6%) was a gift supplied by Aljazerah Industry from Auro laboratories company (India). Four strengths of MET hydrochloride were purchased from the local pharmacies in Saudi Arabia. The origin of these pharmaceutical formulations was Oman, Saudi Arabia, and France with strengths labelled to containing 500, 750, 850, and 1000 mg MET hydrochloride, respectively.

### 2.3. Preparation of Standard and Sample Solutions

Stock standard solutions (1 × 10^−1^ mol L^−1^) of MET were prepared by dissolving an accurately weighed amount (1.66 g) of the standard material in 100 mL of deionized water. These stock solutions were stable for at least two weeks when kept in a refrigerator at 5°C. The working solutions were prepared by diluting stock solution with deionized water to make different concentrations: 1 × 10^−5^–1 × 10^–2^ mol L^−1^ for MET. Both stock and working solutions were kept in a refrigerator at 5°C.

For the preparation of pharmaceutical formulation sample solutions, three tablets from each of the different studied brands were weighed and finely pulverized. Then, a quantity of 100 mg of the MET from each drug brand powder was transferred into a volumetric flask and dissolved in approximately 100 mL of deionized water, mixed for 15 min, and then sonicated for 30 min. These solutions were then maintained in a refrigerator at 5°C.

### 2.4. General Procedures

In the electrochemical validation of the used method, the MET microchip and reference electrode were immersed in the calibration standards solutions, and the EMV and mV of the cell were recorded versus the concentration of MET. The potentiometric validation studies were performed at room temperature (25 ± 2°C). The calibration curves were obtained by plotting subtract logarithm of concentrations against the cell potential, mV. The quantifications of MET samples were achieved under the same conditions. Then, the sample concentrations were calculated by using the linear equations of the calibration curves of MET.

## 3. Results and Discussion

The metformin-based microchip was characterized in terms of sensitivity, selectivity, effect of pH, and response time and reported in our previous work [23]. The organic layer membrane is frequently employed in chemical electrodes due to its great selectivity, sensitivity, and simplicity. Because of the preceding advantages, a selective microchip electrode was constructed in this work to determine the MET hydrochloride in the solutions. The sensitivity of microchips demonstrates that they have significant merits in detecting MET hydrochloride in solutions and in tablet dosage form. There are numerous advantages to using this method, which are rapid, small size, simple, and costless [23].

### 3.1. Validation of Proposed Assays

#### 3.1.1. Linearity and Sensitivity

**Figure 2:**
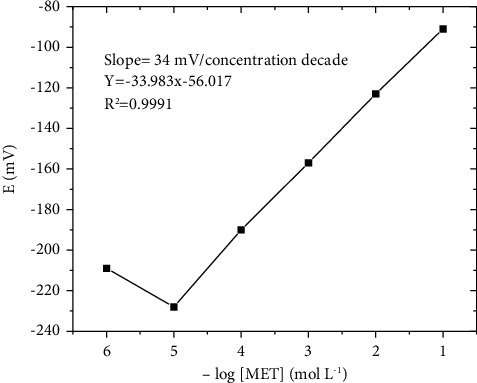
Potentiometric calibration of MET based microchip.

The linearity, selectivity, and sensitivity of metformin hydrochloride are detected by microchips. Calibration graphs were constructed for the detection of MET in aqueous media using a potentiometric microchip (Figure 2). The regression equation of MET was derived, and the results are presented in Table 1. The obtained data shows that the correlation coefficients (*r*^2^) of MET was 0.999. The limits of detection (LOD) and limits of quantification (LOQ) were detected. The LOD and LOQ values of MET were found to be 2.89 × 10^–6^ mol L^−1^ and 8.77 × 10^–6^ mol L^−1^, respectively.

#### 3.1.2. Precision and Accuracy

Replicate analysis of drug solutions at three distinct concentrations was used to assess the precision of potentiometric microchip assays for MET (Table 2). The average relative standard deviation (RSD) of the proposed drug in potentiometric microchips did not exceed 4% for MET (Table 2).

Eventually, the accuracy of the proposed assays was evaluated by determining the recovery percentage of different concentrations. The values presented in the table show that the recovery percentage of all tested drugs was less than 5% (Table 3).

### 3.2. Determination of MET in Pharmaceutical Formulations

Commercially available pharmaceutical dosage forms of MET were analysed using the validated method. The mean percentage recovery relative to the label amounts obtained by previous methods is shown in Table 4. The results indicate that there was no significant difference between the references, label, and recovery which was less than 5% (Table 4).

## 4. Conclusions

This study demonstrates the validation of a recently developed disposable potentiometric microsenor which responsive for the measurement of MET hydrochloride in pharmaceutical formulations for the first time. The potentiometric method depends on tetraphenyl borate: a MET ion pair complex ionophore modified with 5% CNTs sensitive to the MET drug. In addition, this method based on disposable chip assembly, which is used as a low-cost analytical tool (economic), has a rapid response time of less than 10 seconds and is an environmentally friendly “Green” approach. In terms of analytical procedure simplicity, it is a recommended approach for MET hydrochloride and can be employed in high-throughput systems. The proposed approach also offers the merit of determining MET hydrochloride using a single system. These advantages support the use of proposed methodologies as an alternative to current methods in quality control laboratories for regular MET hydrochloride testing.

## Figures and Tables

**Table 1:** Quantitative parameter of linearity.

Parameter	MET
Linear range (mol L^−1^)	0.00001–0.1
Intercept	56.02
Slope	33.98
Correlation coefficient (r)	0.999
LOD, (mol L^−1^)	2.89 × 10^−6^
LOQ, (mol L^−1^)	8.77 × 10^−6^

**Table 2:** MET intra and interday assay precision data (*n* = 3).

Component	Theoretical concentration (mol L^−1^)	Measured conc. (mol L^−1^), RSD (%)	
		Intra-day	Inter-day
MET	0.0002	0.000205 (3.45)	0.000209 (3.73)
	0.002	0.00206 (1.41)	0.00209 (3.06)
	0.02	0.0202 (1.78)	0.0206 (1.17)

**Table 3:** MET % recovery studies and % RSD (*n* = 3).

Component	Concentration, (mol L^−1^)	% recovery (average)	SD × 10^−4^	% RSD
MET	0.0002	102.56	0.071	3.45
	0.002	103.13	0.29	1.41
	0.02	100.97	3.6	1.78

**Table 4:** Metformin hydrochloride in commercially available pharmaceutical formulations data (*n* = 3).

No.	Commercially available pharmaceutical formulations	Origin	Weight of tablet (gm)	Added (nominated) value (mg)	Measured value (mg)	Recovery (%)
1	Tablet, 500	Oman	0.602	83.0	74.5	89.7
2	Tablet, 750	Saudi Arabia	1.093	68.0	68.5	100.7
3	Tablet, 850	France	0.897	94.0	91.1	96.9
4	Tablet, 1000	France	1.071	93.4	88.6	94.8
Average recovery						95.5

## Data Availability

The data used to support the findings of this study are included within the article.
